# Traveler Pro-social Behaviors at Heritage Tourism Sites

**DOI:** 10.3389/fpsyg.2022.901530

**Published:** 2022-06-10

**Authors:** Peng Zhu, Xiaoting Chi, Hyungseo Bobby Ryu, Antonio Ariza-Montes, Heesup Han

**Affiliations:** ^1^College of Hospitality and Tourism Management, Sejong University, Seoul, South Korea; ^2^School of Tourism and Geography Science, Qingdao University, Qingdao, China; ^3^Food Franchise Department, College of Health Sciences, Kyungnam University, Changwon-si, South Korea; ^4^Social Matters Research Group, Universidad Loyola Andalucía, Córdoba, Spain; ^5^College of Hospitality and Tourism Management, Sejong University, Seoul, South Korea

**Keywords:** **COVID**-related heritage tourism, norm activation model (NAM), attitude toward the pro-social behavior, anticipated emotions, social norm, pro-social behavioral intentions

## Abstract

This study aimed to explain the development of tourists’ pro-social intentions during heritage tourism within the pandemic context by combining the norm activation model (NAM) and two significant variables in the theory of planned behavior (TPB). The quantitative data analysis results indicated that the proposed hypotheses have been partially supported, which resonated and enriched the existing studies on COVID-19-related pro-social tourism and tourist behaviors from a theoretical angle. Based on the research outcomes, the corresponding managerial implications for heritage tourism practitioners and meaningful references for future researchers to promote sustainable and pro-social heritage tourism products have been discussed.

## Introduction

Sustainable development has become a new challenge for human life today in all areas due to the negative impacts of the COVID-19 pandemic. The widespread global outbound restrictions resulted in a rapid decline in tourism arrivals and receipts, and heritage tourism has also been severely affected by the current severe situation (UNWTO, 2021). The tourism industry, as an essential part of society, should seek a new normal model to deal with the crisis that the pandemic has brought to humanity, communities, and society (Bae and Chang, 2020). As the pandemic crisis continues to unfold, heritage tourism is experiencing a very difficult time in history with total or partial closures, which have taken a heavy toll on socio-economic development, the cultural life of nations and communities, and the dissemination of knowledge (UNESCO, 2021).

Numerous studies in the field of hospitality and tourism industry have been actively investigating the solutions for the sustainable and pro-social transformation of post-pandemic tourism activities, within the contexts of festival tourism and rural tourism (Chi and Han, 2021; Chi et al., 2021a). Likewise, the investigation of the sustainability of heritage tourism is also a hot research topic (Mohanty et al., 2020; Zhang et al., 2020; Zheng et al., 2020; Hosseini et al., 2021; Singh, 2021). In the social dilemma of the pandemic, tourists are more willing to consume tourism products and services that are beneficial to their physical and mental health (Koh, 2020; Singh, 2021). Most of the heritage tourists are also preferring to visit some sites with a secure travel environment and experience the authentic local culture, traditions, and activities (Park et al., 2019; Chi and Han, 2020; Wang et al., 2021; Kunasekaran et al., 2022). With the implementation of different government policies, worldwide vaccine distribution, and collaborative efforts of the public and private sectors, international and domestic tourism has restarted, and these inclusive, renewable, and sustainable practices will become mainstream alternatives to tourism (Spenceley, 2021). Therefore, the heritage tourism industry has also ushered in new challenges and opportunities for the sustainability of heritage site management, and transformation plays a crucial role in achieving social development, cultural exchange, economic recovery, environmental resource protection, and management (UNESCO, 2021).

As various forms of tourism products and services are adjusted and upgraded, and the perceived knowledge about COVID-19 is becoming more abundant, increasing demand for cultural tourism seems to be emerging. This means that heritage site managers work responsibly with national public health authorities to achieve synergy by promoting new normal lifestyles. The previous research studies on post-pandemic-related pro-social behavior already declared that advocating for the adoption of personal and social norms for pro-social behaviors (such as wearing masks, maintaining social distancing, etc.) in tourist areas is necessary (Chi et al., 2021a,b). All travelers are encouraged to visit and experience heritage sites with different forms of destinations and cultural activities in a pro-social manner to provide a safe and relaxed environment for the travelers and the local communities. Therefore, it is a well worth discussing topic to mobilize people to visit heritage sites in order to get rid of the current social dilemma and to achieve the sustainable transformation of society, which could contribute to a creative new model for the heritage tourism industry in the era of sharing economy and culture in general.

In the existing literature on COVID-19 tourism, some scholars have conducted research to evaluate consumers’ travel attitudes and choices, as well as pro-social behaviors, by integrating the NAM (Zhu and Deng, 2020; Chi et al., 2021a,b; Rather, 2021). In addition, the importance of establishing personal norms and adhering to social norms has also been emphasized in the previous studies (Chi et al., 2021a,b). Therefore, for the sake of human wellbeing and social sustainability, the currently available information about the conformity of travelers’ visit behavior and experiential activities at the heritage sites to the personal norms and social norms in the background of the pandemic is of widespread global concern. The relationships between problem awareness, ascription of responsibility, anticipated feelings, attitude toward the pro-social behavior, social norm, personal norm, and behavioral intentions were investigated in the former pro-environmental/pro-social-related studies (Han, 2014; Kim and Hwang, 2020; Chi et al., 2021a,b). To the best of our knowledge, the researchers have not adequately explored these types of significant connections in the background of COVID-19 heritage tourism to measure heritage visitors’ response efforts and the pro-social behavioral intentions during the pandemic.

Therefore, the research objectives are listed as follows: (1) investigate the complicated relationships between variables based on the NAM to predict tourists’ pro-social behavioral intentions in the heritage tourism post-pandemic, (2) establish the importance and mediating effects of dimensions in the proposed model of the development of pro-social behavioral intentions via personal norm, and (3) deepen the theoretical framework by taking into account the moderating effect of gender on the role of pro-social behavioral intentions in the heritage tourists.

## Literature Review

### Pro-social Heritage Tourism in the With-Corona Era

**FIGURE 1:**
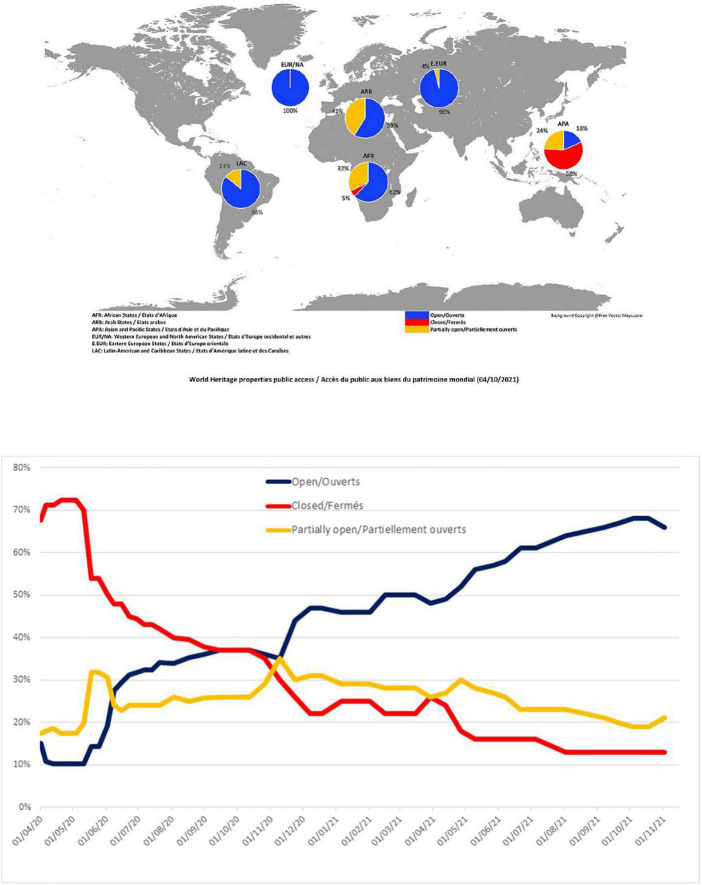
The operating states of world heritage sites in a global scope. Sources from: https://en.unesco.org/covid19/cultureresponse/monitoring-world-heritage-site-closures.

Culture tourism is one of the biggest tourism markets, which continued to expand by about 15% every year before COVID-19, and it was three times faster than mass tourism (Huibin et al., 2012; Zhao et al., 2020). Heritage tourism is an essential component of the global tourist business in the current tourism market, which also accounts for a major portion of cultural tourism (Poria et al., 2003; Richards, 2018). As a result of the COVID-19 pandemic outbreak, the impact is felt in all areas of life, which include health, society, and economic growth (Khan et al., 2020; Lenzen et al., 2020; Roy, 2020). Unsurprisingly, the heritage tourism industry has also been suffering uncertainness and new challenges within a global scope, due to the complete and partial closure of the heritage sites (UNESCO, 2021), which is exhibited in Figure 1. The travel restrictions have largely cut off the flow of tourists and led to a crisis for related income sources of heritage sites and local communities. Although the reduction in the number of tourists releases ecological pressures on some natural heritage sites to some extent, it has simultaneously brought negative social and economic impacts on local communities, such as uncertain livelihoods, increased poverty, reduced cultural conservation, and the ignored protection and maintenance management of cultural heritage sites (Murzyn-Kupisz, 2012; Xie et al., 2020; Goretti et al., 2021). Therefore, heritage tourism was important for promoting development in economic, social, and environmental terms (Hartmann, 2020; Lak et al., 2020).

**FIGURE 2:**
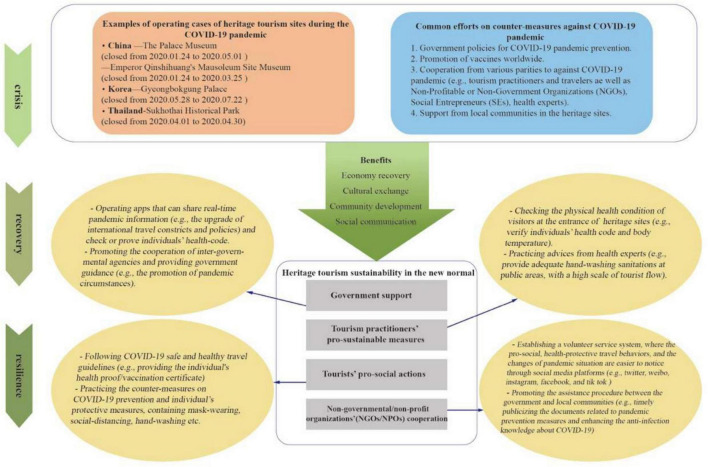
Heritage tourism sustainability in the new normal. Sources from: Emperor Qinshihuang’s Mausoleum Site Museum Website (2020), Gyeongbokgung Palace Management Office Website (2020), REUTERS (2020), The Palace Museum Website (2020), Tian (2020), and Spenceley (2021).

It is the common pursuit of the tourists, governments, and non-governmental organizations to help relaunch heritage sites with safer and easier experiences. Multi-party cooperation facilitates the vitality of economic, cultural, and community partners to make heritage tourism sustainable (Singh, 2021). In addition, some scholars have discussed pro-social behaviors and sustainability studies in previous studies about hospitality and tourism (Han et al., 2019; Kim and Hwang, 2020; Chi et al., 2021a,b). However, few current studies have explored pro-social behaviors and sustainability in the context of heritage tourism. Considering the fact that it is challenging to manage the tourists’ mobility and daily practices in terms of wearing masks, social distancing, and sanitation maintenance, it is essential for the heritage site management department to properly guide and mobilize tourists to work together in order to create a pro-social and secure travel environment in the post-pandemic era. Therefore, investigating tourists’ perceptions and behaviors about participating in the actions of creating a pro-social heritage travel environment and exploring what kind of alternatives to develop pro-social and sustainable heritage tourism products within the crisis brought by COVID-19 are the imperative subjects (Higgins-Desbiolles, 2020; Yeh, 2020). As exhibited in Figure 2.

### Norm Activation Model

Schwartz (1977) described that the normative activation model (NAM) is a vital model developed in the background of altruistic behavior. The model was designed to explain the individuals’ pro-social or pro-environmental behavioral intentions, and it has also been used in recent studies on hospitality and tourism (Kim and Hwang, 2020; Chi et al., 2021a,b). The NAM predicts pro-social or pro-environmental behavior by utilizing three antecedents, which include problem awareness, ascription of responsibility, and personal norms, and it is the primary construct where the individuals engage in pro-social or pro-environmental behavior (Schwartz and Howard, 1981; Kim and Hwang, 2020), and the development of pro-social behavior intentions are directly or indirectly mediated by the ascription of responsibility and personal norms (Chi et al., 2021a). Personal norms are the closest antecedents when predicting pro-social or pro-environmental intentions/behaviors in the NAM (Onwezen et al., 2013; Chi et al., 2021a,b). The terms the personal norm, moral norm, and moral duty are frequently used interchangeably in the existing literature (Han and Hyun, 2017). In this theory, the process of norm activation begins with an individual’s awareness of the potentially negative repercussions, which triggers a sense of responsibility for the negative effects of not adopting pro-social/environmental behavior (Schwartz, 1977), and this awareness stimulates personal norms, which determines whether he or she should engage in or avoid a specific behavior to prevent a negative outcome (Han, 2014; Kim and Hwang, 2020).

The existing research on the NAM-based theoretical framework has been widely used to understand the pro-social/pro-environmental behavior/intention of the hotel and tourism industry (Han et al., 2019; Kim and Hwang, 2020; Chi et al., 2021a,b). Specifically, Han et al. (2019) suggested that the variables of anticipated emotional, personal attitudes and social norms play a significant role in explaining the decision-making process involved in the consumer purchase of eco-friendly cruise products. Kim and Hwang (2020) used two theories to explain the creation of behavioral intentions for drone food delivery services in a pro-environmental background: the normative activation model (NAM) and the Theory of planned behavior (TPB). Chi et al. (2021a) effectively expanded the normative activation framework in festival tourism in the background of COVID-19, which once again verified its complex relationship with the NAM and explained the pro-social behavioral intentions of festival tourists. Whether in the field of hotel or tourism, the NAM has been proven to be an effective measurement model of pro-social/pro-environmental behavior/intention (Han, 2014; Kim and Hwang, 2020; Chi et al., 2021b). Every heritage tourist should be aware of the seriousness of the COVID-19 problem when visiting the heritage site, that everyone has a potential responsibility for pandemic prevention and that the individuals must regulate their behavior. As such, the NAM was utilized to explain the pro-social behavioral intentions in the COVID-19-related heritage tourism setting, and the following research hypotheses were developed:

**H1.** Problem awareness regarding COVID-19 influences the ascription of responsibility among the heritage travelers.

**H2.** Ascription of responsibility influences personal norms among heritage travelers.

**H3.** Personal norm influences pro-social behavioral intentions among heritage travelers.

### Anticipated Feelings of Pride and Guilt

Anticipated emotions are frequently mentioned in NAM research. Anticipated emotions include anticipated feelings of pride and anticipated feelings of guilt, which are frequently seen as self-conscious feelings and are particularly important in understanding the pro-social or pro-environmentally responsible decision-making behavior within the NAM (Han et al., 2017; Chi et al., 2021a). In the existing studies, the moral norm is closely related to the anticipated positive and negative emotions. When evaluating certain individual behaviors related to pro-social and pro-environment, the anticipated emotion of pride inspires compliance with the moral norm, and an anticipated emotion of guilt promotes individuals to avoid violating a moral norm (Schwartz, 1977; Han, 2014). Numerous studies have pointed out that the anticipated emotions of pride and guilt could be well integrated with the NAM in different contexts (Onwezen et al., 2013; Han et al., 2017). The anticipated feelings of pride and guilt directly affect personal norms as emotional triggers, thereby triggering pro-social/environmental behaviors. The study findings showed the existence of the mediating relationship between anticipated emotions and NAM variables (Han, 2014; Chi et al., 2021a). Therefore, we employed the NAM as our basic framework, and the anticipated emotional constructs were integrated in the basic framework to examine the following related hypothesized associations.

**H4.** The anticipated feeling of pride influences personal norms among heritage travelers.

**H5.** The anticipated feeling of guilt influences personal norms among heritage travelers.

### Theory of Planned Behavior

Due to its powerful predictive power, the theory of planned behavior (TPB) is often used in social psychology to predict human decisions or behavior (Rivis et al., 2009; Sommer, 2011). It is an extended theoretical model of the theory of reasoned action (TRA) (Ajzen, 1991). TPB has also been successfully applied to tourism and hospitality research with regard to the eco-friendly decision-making process (Han and Hyun, 2017; Kim and Hwang, 2020; Chi et al., 2021b). In the study of pro-social behavior in tourism and hospitality, many researchers have explored extended theoretical models for combining NAM and TPB to predict the development of pro-social behavior, within the contexts of festival tourism, cruise industry, drone food delivery services, etc. (Han and Hwang, 2016; Kim and Hwang, 2020; Chi et al., 2021b), in which two important elements of the TPB theory factors (i.e., attitude toward the behavior and social norm) are repeatedly emphasized when combined with NAM (Chi et al., 2021a,b; Kim and Hwang, 2020). However, little research has been done on integrating the TPB and the NAM in heritage tourism during the post-pandemic era. Therefore, it is essential to extract the significant variables of the NAM and TPB to predict the development of tourists’ pro-social behavioral intentions while visiting a certain heritage tourism destination in the post-pandemic era.

### Attitude Toward the Pro-social Behavior

At present, the prior studies have discussed the NAM and the TPB together to analyze tourists’ attitudes toward pro-social behaviors in various contexts (Han and Hyun, 2017; Kim and Hwang, 2020; Chi et al., 2021a). The current study also integrated the NAM with two important variables of the TPB (i.e., attitude toward the pro-social behavior and subjective norm). Attitudes toward behavioral intentions are frequently discussed in the literature on pro-social/pro-environmental decision-making processes and behaviors, which include the studies by Bamberg and Möser, 2007; Han and Hyun, 2017, and Ritchie et al. (2021). Attitude toward the behavior is broadly defined as “the extent to which an individual’s evaluation or appraisal of the act is favorable or unfavorable” (Ajzen, 1991). Attitudes toward the behavioral importance have been repeatedly emphasized in all the studies conducted on theories/models that are based on pro-social motivations, which include NAM, in order to investigate the impact of attitudes toward a certain behavior on the function of the personal norm in the decision-making process of adopting the pro-social/pro-environmental behavioral intentions (Morren and Grinstein, 2021). In the study of Thøgersen and Ölander (2006), the understanding of the relationship between attitude, norm, and behavior was further deepened by analyzing the interaction between attitude variables and eco-friendly purchase behaviors. In recent studies, the driving role of attitudes toward the behavior to arouse personal norms and activate pro-social and pro-environment behavioral intentions has also been continuously recognized (Kim and Hwang, 2020; Morren and Grinstein, 2021). Therefore, we proposed the following hypothesis in the background of heritage tourism:

**H6.** Attitude toward pro-social behavior influences personal norms among heritage travelers.

### Social Norm

Subjective norms can be alternatively termed as social norms, with a definition of “perceived societal pressure to do or refrain from performing the action” (Ajzen, 1991; Chi et al., 2021a). The concept of social norms has been extensively studied to explore the influence that normative individual decision-making processes and the pro-social/pro-environmental behavioral intentions have (Budovska et al., 2020; Chi et al., 2021a). Empirical evidence provided by the recent studies in the field of hospitality and tourism suggests that social norms are widely recognized by many researchers as valid predictors of personal norms and pro-social/pro-environmental behavioral intentions (Han et al., 2019; Kim and Hwang, 2020; Chi et al., 2021a). According to Han et al. (2019), social norms play a prominent role in the decision process of buying eco-cruise products, which verified its direct and indirect positive effects on the environmentally buying intentions and personal norms. Similarly, Kim, and Hwang (2020) also asserted that a significant association existed between social norms and personal norms and pro-environmental behavioral intentions in the drone delivery services environment. Chi et al. (2021a) established that a person’s moral norm is influenced by societal pressure and that this relationship stimulates the intention to behave pro-socially. Therefore, we proposed the following hypotheses in the background of COVID-19 heritage tourism:

**H7.** Social norm influences personal norms among heritage travelers.

**H8.** Social norm influences pro-social behavioral intentions among heritage travelers.

### Moderator of Gender

**FIGURE 3:**
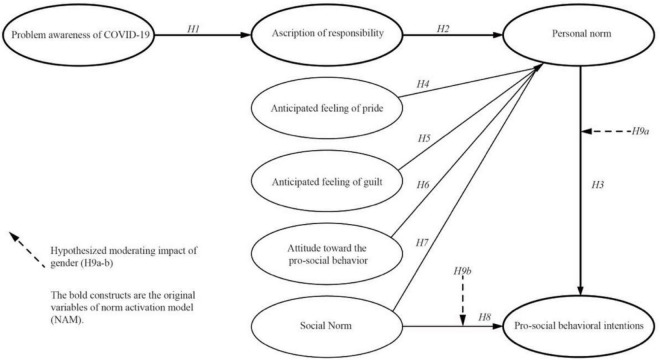
Proposed conceptual model.

Researchers pay particular attention to the factors in the study of demographics because they affect behavioral intentions, and gender is regarded as one of the moderators that influences an individual’s development of pro-society/pro-environmental and pro-sustainable intentions and actions (Chi et al., 2021a). Gender differences could lead to different values and social expectations of certain intentions and behaviors due to the differing cultural and psycho-metric backgrounds (Han et al., 2018). In the existing tourism studies, Han et al. (2018) found a gender difference in the tourist’ pro-environmental actions, such as waste reduction and recycling. Specifically, the pro-social behavioral intentions of men are more likely to trigger than women on driven by social norms. A most recent COVID-related festival tourism by Chi et al. (2021a) has utilized the NAM to evaluate the moderating impact of gender on the association between personal norms and pro-social behavioral intentions, which indicated that women were more likely to be driven by moral norms to adopt pro-social behavioral intentions, such as wearing masks, keeping social distance, and practicing sanitation. Therefore, the following hypotheses were proposed, and Figure 3 shows the proposed conceptual model.

**H9a.** The impact of the personal norms on pro-social behavioral intentions significantly differs across male and female heritage travelers.

**H9b.** The impact of the social norms on pro-social behavioral intentions significantly differs across male and female heritage travelers.

## Methodology

### Measures and Data Collection

The measurement items that were designed for the eight study constructs in this current research were derived from existing studies regarding pro-sustainable tourism and pro-environmental behavior intentions (Ajzen, 1991; Han, 2014; Han and Hyun, 2017; Han et al., 2019; Kim and Hwang, 2020; Chi et al., 2021a,b). Specifically, our research to integrate and strengthen the NAM theory, including problem awareness, ascription of responsibility, personal norm, anticipated feelings, attitude toward the pro-social behavior, and societal norms, were cited from the currently existing research. More specifically, problem awareness regarding COVID-19 was measured using three items adapted from Han (2014) and Kim and Hwang (2020). Moreover, the ascription of responsibility was measured with three items used by Han (2014) and Kim and Hwang (2020). Personal norm was measured with three items borrowed from Han and Hyun (2017) and Han et al. (2019). Anticipated feelings of pride and guilt were measured with three items separately (Han, 2014; Chi et al., 2021a). Measurement items for some important structures of TPB, such as attitudes, social norms, and behavioral intentions, were borrowed from previous studies. Specifically, four items regarding attitude were extracted from Ajzen (1991); Han and Hyun (2017), and Kim and Hwang (2020). Three items on social norms were adopted from Han and Hyun (2017); Kim and Hwang (2020), and Chi et al. (2021b). Lastly, six items regarding the use of behavioral intentions were obtained from Han and Hyun (2017) and Chi et al. (2021a). All the study items employed slightly modified measures to fit the present research setting of a pro-social heritage tourism study. This study brought together graduate students majoring in hospitality, and a focus group of tourism scholars and hospitality industry experts to modify this measurement methodology process. After that, the study variables were then assessed using a seven-point Likert scale ranging from (1) strongly agree to (7) strongly disagree. The problem awareness regarding COVID-19, the ascription of responsibility, personal norm, social norm, pro-social behavioral intentions, and anticipated feelings of pride and guilt were assessed using three items, while attitude toward the pro-social behavior was evaluated utilizing four items. The scholars thoroughly reviewed the survey questions (see Appendix Table A1).

The study adopted an online survey that was conducted in China after the COVID-19 outbreak. The participants participated in the questionnaire survey through Chinese social media apps, such as WeChat, Weibo, etc. For the sake of clearly explaining the role of pro-social behavior, the respondents in this study were first introduced to the idea of pro-social behavior, and only those who had visited the heritage sites at least once were invited to take part in the survey during the COVID-19 pandemic. In this study, 379 valid questionnaires were used for the subsequent analysis process through SPSS and AMOS 23 software. The sample profiles are summarized in Table 1.

**TABLE 1:** Demographic characteristics of the respondents.

Variable	Category	Distribution	Valid percentage
Gender	Female	154	40.6
	Male	225	59.4
Age	Mean	29.4	
Occupation	Service/salesperson	41	10.8
	Official from government	31	8.2
	Technician/academician	53	14
	Student	130	34.3
	Others	124	32.7
Marital status	Married	189	49.9
	Single	171	45.1
	Other	19	5.0
Average monthly income	Under 5000 RMB	178	47
	5001-10000 RMB	135	35.6
	10,001-15000 RMB	52	13.7
	Over 15000 RMB	14	3.7
Education	High school or below	34	9.0
	Three-year college	47	12.4
	Bachelor’s degree	226	59.6
	Postgraduate degree	72	19.0

## Results

### Confirmatory Factor Analysis

The confirmatory factor analysis was used to estimate the measurement model, referred to hereinafter as CFA. The AMOS (Analysis of Moment Structures) program was used to assess the suggested conceptual model’s measurement structure. As shown in Table 2, the results of the measurement model of CFA show that the data fits well (χ^2^ = 739.886, *df* = 322, χ^2^*/df* = 2.298, *p* < 0.001, *CFI* = 0.962, *IFI* = 0.963, *TLI* = 0.956, *RMSEA* = 0.059). The factor loadings were all in the range of 0.824–0.974, which were discovered to be statistically significant (*p* < 0.001). The specific variables employed in this analysis and their standardized factor loadings are presented in Table 2. Lastly, the composite reliability values ranged from 0.917 to 0.963, which indicated a high level of internal consistency for each construct, because the composite reliability values were above the minimum threshold value of 0.60 (Bagozzi and Pieters, 1998). The squared correlations between the variables ranged from 0.708 to 0.897 and are presented in Table 2. For all the study variables, the AVE values exceeded the recommended cut-off of 0.50 (Fornell and Larcker, 1981), and the results suggested that the square roots of AVE values were larger than the correlation values of the constructs, which implied the establishment of discriminant validity (Fornell and Larcker, 1981), and results are displayed in detail in Table 3.

**TABLE 2:** Measurement items and results of confirmatory factor analysis.

Measures	Factor loading	CR	AVE
Problem awareness of COVID-19 (PAC)		0.921	0.796
PAC1	0.894		
PAC2	0.876		
PAC3	0.906		
Ascription of responsibility (AR)		0.939	0.838
AR1	0.921		
AR2	0.898		
AR3	0.928		
Personal norm (PN)		0.937	0.832
PN1	0.923		
PN2	0.903		
PN3	0.910		
Anticipated feeling of pride (AFP)		0.917	0.787
AFP1	0.902		
AFP2	0.874		
AFP3	0.885		
Anticipated feeling of guilt (AFG)		0.963	0.897
AFG1	0.959		
AFG2	0.934		
AFG3	0.948		
Attitude toward the pro-social behavior (APB)		0.949	0.822
APB1	0.897		
APB2	0.903		
APB3	0.907		
APB4	0.920		
Social norm (SN)		0.924	0.802
SN1	0.974		
SN2	0.848		
SN3	0.860		
Pro-social behavioral intentions (PBI)		0.936	0.708
PBI1	0.862		
PBI2	0.866		
PBI3	0.838		
PBI4	0.824		
PBI5	0.826		
PBI6	0.833		
***Goodness-of-fit statistics:*** χ^2^ = 739.886, df = 322, χ^2^/df = 2.298, *p* < 0.001, CFI = 0.962, IFI = 0.963, TLI = 0.956, RSMEA = 0.059.			

*CR, Composite Reliability; AVE, Average Variance Extracted.*

**TABLE 3:** Correlations and values of square root of AVE among model constructs.

Constructs	PAC	AR	PN	PBI	APB	AFP	AFG	SN
PAC	**0.892**							
AR	0.589	**0.915**						
PN	0.387	0.317	**0.912**					
PBI	0.408	0.278	0.321	**0.842**				
APB	0.345	0.254	0.715	0.801	**0.907**			
AFP	0.348	0.285	0.638	0.727	0.631	**0.887**		
AFG	0.256	0.306	0.321	0.312	0.299	0.400	**0.947**	
SN	0.255	0.314	0.339	0.348	0.345	0.466	0.844	**0.896**

*PAC, problem awareness of COVID-19; AR, ascription of responsibility; PN, personal norm; PBI, pro-social behavioral intentions; APB, attitude toward the pro-social behavior; AFP, anticipated feeling of pride; AFG, anticipated feeling of guilt; SN, social norm.*

*The values of square root of AVE are in the diagonal (bold).*

### Structural Equation Modeling

**FIGURE 4:**
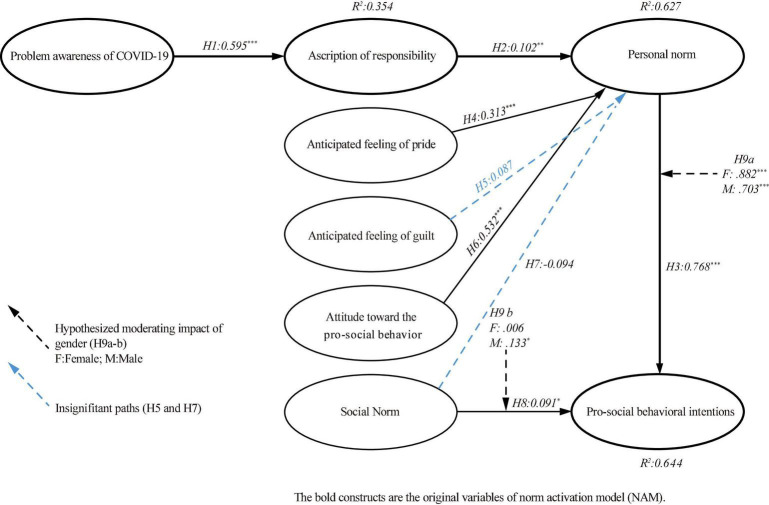
Results of the structural equation model.

The results from the structural equation model (SEM) are shown in Figure 4 and Table 4. The data is wellsuited to the proposed structural model (Goodness-of-fit statistics*:*χ^2^ = 891.895, *df* = 332, χ^2^*/df* = 2.686, *p* < 0.001, *CFI* = 0.950, *IFI* = 0.950, *TLI* = 0.943, and *RMSEA* = 0.067). The results of the SEM are presented in Table 4. Hypotheses 1, 2, and 3 suggested the existence of relationships between the NAM’s original constructs. The results of the study found that the problem awareness of COVID-19 significantly affected the attribution of responsibility (β = *0.595 and p* < 0.01). The personal norm was positively influenced by ascription of responsibility (β = *0*.102 *and p* < *0.01)*, and personal norm had a significant effect on pro-social behavioral intentions (β = 0.768 *and p* < 0.01). Therefore, the results of Hypotheses 1, 2, and 3 were supported. The proposed relationships among attitudes toward the pro-social behavior, anticipated feelings of guilt and pride, social norm, and personal norm were evaluated. The study result showed that anticipated feeling of pride (β = 0.313 and *p* < 0.01) and attitude toward the pro-social behavior (β = 0.532 and *p* < 0.01) showed a positive and significant effect on the personal norms. Thus, Hypotheses 4 and 6 were supported. However, anticipated feelings of guilt (β = 0.087 and *p* > 0.05) and social norm (β = −0.094, *p* > 0.05) did not have a significant influence on the personal norm. Therefore, Hypotheses 5 and 7 were not supported. The findings showed that social norms influenced the pro-social behavioral intention (β = 0.091, *p* < 0.05), which supported Hypothesis 8. Table 4 displays the results of the SEM.

**TABLE 4:** Hypotheses testing and the structural model outcomes.

Hypothesized paths		Standardized coefficients	*t*-values	Results
Hypothesis1	PAC→AR	0.595	12.008***	Accepted
Hypothesis2	AR→PN	0.102	2.613**	Accepted
Hypothesis3	PN→PBI	0.768	16.076***	Accepted
Hypothesis4	AFP→PN	0.313	5.814***	Accepted
Hypothesis5	AFG→PN	0.087	1.194	Rejected
Hypothesis6	APB→PN	0.532	10.367***	Accepted
Hypothesis7	SN→PN	–0.094	–1.227	Rejected
Hypothesis8	SN→PBI	0.091	2.350*	Accepted
***Total variance explained:*** R^2^ of AR = 0.354 R^2^ of PN = 0.627 R^2^ of PBI = 0.644				
***Goodness-of-Fit Statistics:*** χ^2^ = 891.895, df = 332, χ^2^/df = 2.686, *p* < 0.001, CFI = 0.950, IFI = 0.950, TLI = 0.943, RSMEA = 0.067.				

PAC, problem awareness of COVID-19; AR, ascription of responsibility; PN, personal norm; PBI, pro-social behavioral intentions; AFP, anticipated feeling of pride; AFG, anticipated feeling of guilt; APB, attitude toward the pro-social behavior; SN, social norm. *p < 0.05, **p < 0.01, ***p < 0.001.

### Structural Invariance Testing

The 379 valid responses were divided into two groups comprising male and female participants. There were 225 cases in the male group and 154 cases in the female group. The results of baseline model with these two groups were very satisfactory (χ*^2^* = 1276.650, *df* = 664, χ*^2^/df* = 1.923, *p* < 0.001, *RMSEA* = 0.049, *CFI* = 0.945, *IFI* = 0.946, and *TLI* = 0.938). The nested models and the baseline model were then compared using a chi-square difference test, and between gender groups, the specific relation of interest between these two groups was equally restricted. As shown in Figure 4 and Table 5, according to the findings, the relationship between the personal norm and pro-social behavioral intentions (△χ*^2^* [1] = 4.305, *p* < 0.05), and between the social norm and pro-social behavioral intentions (△χ*^2^* [1] = 3.029, *p* < 0.05) were significantly different across male and female heritage travelers, so Hypotheses 9a and 9b were supported.

**TABLE 5:** Results of the structural invariance models.

		Female			Male	Baseline model	Nest model
Paths		β	t-value	β	t-value	freely estimated	equally restricted
H9a	PN→PBI	0.882	10.788***	0.703	11.602***	χ^2^ (664) = 1276.650	χ^2^ (665) = 1280.955
H9b	SN→PBI	0.006	0.111	0.133	2.518*	χ^2^ (664) = 1276.650	χ^2^ (665) = 1279.679
**Baseline model Goodness-of-fit indices:** χ^2^ = 1276.650, df = 664, χ^2^/df = 1.923; p < 0.001, CFI = 0.945, IFI = 0.946, TLI = 0.938, and RMSEA = 0.049.								
**Chi-square difference test:**								
Δχ^2^ (1) = 4.305, p < 0.05			H9a: supported					
Δχ^2^ (1) = 3.029, p < 0.05			H9b: supported					

Structural invariance models for women (N = 154) and men (N = 225). PN, personal norm; SN, social norm; PBI, pro-social behavioral intentions. *p < 0.05, **p < 0.01, ***p < 0.001.

## Discussion and Implications

### Theoretical Implications

The investigation of the role of the new normal in assessing tourists’ pro-social intentions and achieving the sustainability of the tourism industry has become increasingly vital during and after the COVID-19 pandemic (Chi et al., 2021a). However, few studies have been devoted to the role of heritage tourism’s pro-social behavior intentions and tourism sustainable development in the context of post-pandemics. From the theoretical aspect, the development process of the pro-social behavioral intentions and tourism sustainability was first described in the context of post-pandemic heritage tourism, and this research echoed the previous studies on pro-social behavior and sustainability (Han et al., 2019; Kim and Hwang, 2020; Chi et al., 2021a,b). The study enriched the current COVID-19 tourism research, focusing on tourists’ moral norms and pro-social and pro-sustainable behavioral intentions during the post-pandemic period, which provides a critical theoretical basis for developing or adjusting pro-social and pro-sustainable tourism products in the post-pandemic era.

Consistent with the previous studies (Kim and Hwang, 2020; Chi et al., 2021a), NAM was successfully used to illustrate the formation of the pro-social behavioral intentions in the background of heritage tourism in the post-pandemic period. The variables of problem awareness, ascription of responsibility, and anticipated feeling of pride were efficiently examined as the contributors to promoting personal norms to practice pro-social/pro-environmental behaviors. It is worth mentioning that the study also combined the two important key concepts of TPB (i.e., attitude toward the behavior and social norm) with an extended NAM, to strengthen the theoretical framework and analyze the decision-making process of tourists’ pro-social behavioral intentions (Thøgersen and Ölander, 2006; Han and Hyun, 2017; Kim and Hwang, 2020; Morren and Grinstein, 2021). Moreover, the mediating role of the research variables (i.e., ascription of responsibility, personal norm, and pro-social behavioral intentions) was confirmed within the proposed model, which was consistent with the results of previous studies that when the travelers develop pro-social behavioral intentions, they were directly and/or indirectly affected by these factors (Han et al., 2017; Chi et al., 2021a). Our research results have also proved and supported that the extended NAM model was more efficiently used to analyze the formation process of the pro-social behavioral intentions of heritage tourists and provided significant theoretical value for the pro-social and pro-sustainable heritage tourism industry.

Generally, numerous researchers agreed that social norms could directly or indirectly influence personal norms and pro-social/pro-environmental behavioral intentions in the previous studies (Han and Hyun, 2017; Kim and Hwang, 2020; Chi et al., 2021a). Non-etheless, this study differed from the previous studies that the personal norms were not positively affected by the social norms and that the social norms have a direct impact on pro-social behavioral intentions in the COVID-19 pandemic context of heritage tourism. In other words, social pressure does not play an important role in moral obligation. This indicates that when others say that pro-social behavior, such as wearing-mask, washing hands frequently, and keeping distance, is important, they are not likely to be socially pressured to act in a certain way. Moreover, the anticipated feeling of guilt has not stimulated the personal norm to induce tourists to take pro-social behavioral intentions. It can be inferred that the anticipated feeling of guilt cannot be a key concept in the background of heritage tourism in the post-pandemic period, which is consistent with Schwartz (1977). The study showed that when the personal norm was added in the multiple regression analysis, the effect of anticipated guilt on behavior was no longer significant for a variety of pro-social actions. Therefore, this study provides evidence for the relationship between these key structures in the heritage tourism context during the COVID-19 pandemic, which also enriches the sustainability of the literature.

Meanwhile, our study verified the moderating impact of gender factors on the connection between individual behavioral norms and societal behavioral norms in forming pro-social behavioral intentions. In the study of individual pro-environmental/pro-social behavioral differences, gender was a significant moderating influence, consistent with the previous results (Han et al., 2018; Chi et al., 2021a). Women were more likely to develop pro-social behavioral intentions driven by personal norms, but in terms of social norms, men were more likely than women to develop pro-social behavioral intentions, which was reflected in health protection, such as maintaining social distancing and conducting hygiene activities to avoid infection during the pandemic. The differences in activation with respect to individual behavior and social behavior in the practice of pro-social activities were further validated by examining the moderators of gender in an extended conceptual model, which provides new perspectives regarding the demographic and psychological predictors of the study of individual normative behavior, social normative behavior, and pro-social behavior in different domains.

### Managerial Implications

The proposed research model based on the NAM has been successfully applied in the context of heritage tourism, where the personal norm was positively and significantly correlated with the individuals’ pro-social behavioral intentions. For instance, in the heritage tourism domain post-pandemic, the individuals’ moral obligation played a crucial factor in triggering the pro-social behavioral intentions. Therefore, the government can share real-time COVID-19 pandemic information by checking/certifying an individual’s healthy code through apps, such as WeChat, Alipay, Kakaotalk, or Uber.

The management of the pandemic can be facilitated by promoting the cooperation between the inter-governmental agencies and providing government guidance, such as giving continuous updates about the pandemic situation and the promotion of pandemic circumstances. Various apps that can share real-time pandemic information (e.g., the upgrade of international travel constricts and policies) and check or prove individuals’ health codes, to provide the heritage site managers and tourists with real-time updates on the pandemic can be operated. Tourism practitioners in heritage sites can correctly guide tourists to take health protection measures to practice pro-social behaviors in accordance with heritage site code of conduct requirements, which can include having the visitors provide proof of COVID-19 vaccination certificate or recovery, and they can also implement the recommendations of hygiene experts to provide adequate hand sanitizer and sanitation facilities in public areas with high tourist traffic at the heritage site’s tourist attractions and check the physical health condition of visitors at the entrance of heritage sites, such as verifying individuals’ health code and body temperature.

In addition, non-government organizations (NGOs) can establish a volunteer service system, where the pro-social and health-protective travel behaviors and the changes in the pandemic situation are easier to notice through platforms such as Weibo, Twitter, Instagram, TikTok, Facebook, etc. The NGOs can also promote the assistance procedure between the government and local communities, such as timely publicizing the documents related to pandemic prevention measures, enhancing the anti-infection knowledge about COVID-19, and improving moral obligation to practice pro-social behaviors. The heritage site managers could think about increasing the collaboration with the heritage site staff, and they can post signs about the social hazards of COVID-19 at the heritage sites, distribute safe and healthy travel guide brochures, and make regular announcements about pandemic-related health precautions and safety protocols, such as wearing masks, using hand sanitizer, and maintaining safe social distancing. They can also default the maximum visits in a certain period, monitor travelers at site entrances for COVID-19 symptoms, such as high temperatures, and require evidence of a negative COVID-19 test result, health travel codes, and the travel vaccination certificate. Additionally, electronic payment methods, including WeChat pay, Alipay, and other smart payments, can be further efficiently used at the heritage sites.

This research revealed that positive anticipatory emotions and attitudes were important drivers of personal norms, which indirectly induced pro-social behavioral intentions. This can be explained by the fact that tourists’ personal moral obligations during heritage tourism activities were strongly influenced by emotions and attitudes. This means that boosting the tourists’ feelings and the attitude toward the personal norm is essential to obtain a thorough understanding of their pro-social decision for visiting heritage sites. Therefore, it is more important for tourism managers to consider taking into account the anticipated emotions and attitudes toward the behavior of tourists when prescribing safety and health precautions at the heritage sites. For example, the tourist guide booklet can use eye-catching pictures, and the official app can be used to promote COVID-19 safety and health knowledge and the regulations in tourism sites, to remind the visitors to observe what behaviors are pro-social, wise, and encouraged, and what behaviors are unfavorable, associated with high infectious risk, and be discriminated against by the public and local communities. These measures can promote the individuals’ moral obligations on preventing pandemic spread by enhancing their anticipated emotions and attitude toward the pro-social behaviors.

Given that gender differences significantly affected the strength of the connection between personal norms and social norms with regard to pro-social behavioral intentions. Men and women exhibited different moral obligations and social norm levels to take pro-social behavioral intentions in the background of the post-pandemic. Therefore, different effective strategies should be developed to induce pro-social behavioral intentions according to the groups of female and male tourists.

## Limitations and Future Research

The current research offers both theoretical and managerial implications, but several limitations need to be carefully illustrated, that is, the survey in this study was limited to just heritage travelers in China, so the later researchers were encouraged to perform cultural and cross-country surveys and apply the proposed framework to other hospitality and tourism fields to examine the broad applicability of our study results. On the other hand, the research scope was merely limited to one foremost and prevalent facet of tourists’ pro-social performance in heritage tourism. Future scholars in the other sectors of tourism can utilize the framework proposed in the current study from other tourism perspectives to explore additional significant aspects that are related to the development of heritage tourists’ pro-social performance while attending heritage tours. Lastly, the demographic characteristics of tourists visiting heritage sites show that there is an imbalance between the male and female participants in the particular background of the COVID-19 pandemic, and later scholars could adopt a database with higher validity to elaborate more comprehensive insights into tourists’ pro-social behavioral patterns, from multiple perspectives such as health psychology and crisis management.

## Data Availability Statement

The raw data supporting the conclusions of this article will be made available by the authors, without undue reservation.

## Author Contributions

All authors contributed to conceptualization, formal analysis, investigation, methodology, writing, and editing of the original draft of the manuscript.

## Conflict of Interest

The authors declare that the research was conducted in the absence of any commercial or financial relationships that could be construed as a potential conflict of interest.

## Publisher’s Note

All claims expressed in this article are solely those of the authors and do not necessarily represent those of their affiliated organizations, or those of the publisher, the editors and the reviewers. Any product that may be evaluated in this article, or claim that may be made by its manufacturer, is not guaranteed or endorsed by the publisher.

## Appendix

**APPENDIX TABLE A1:** Measurement items.

*Problem awareness of COVID-19* (Han, 2014; Kim and Hwang, 2020)
PAC1- The COVID-19 outbreak and pandemic are more serious than what individuals think in the tourism industry
PAC2- I am concerned that COVID-19 and its negative impact on the tourism industry will last longer than I expected
PAC3- I am aware of the seriousness of COVID-19 and its negative influence on the tourism industry
***Ascription of responsibility*** (Han, 2014; Kim and Hwang, 2020)
AR1- Every traveler is partly responsible for the COVID-19 outbreak and pandemic
AR2- Every traveler is jointly responsible for the COVID-19 outbreak and pandemic
AR3- Every traveler must assume responsibility for the COVID-19 outbreak and pandemic
***Personal norm*** (Han and Hyun, 2017; Han et al., 2019)
PN1- I feel morally obliged to practice pro-social behaviors at a heritage site by closely following the COVID-19 safe and healthy travel guidelines for my next vacation trip
PN2- I feel personally obliged to practice pro-social behaviors at a heritage site by closely following the COVID-19 safe and healthy travel guidelines for my next vacation trip
PN3- I feel a moral obligation to engage in pro-social behaviors at a heritage site by closely following the COVID-19 safe and healthy travel guidelines for my next vacation trip
***Anticipated feeling of pride*** (Han, 2014; Chi et al., 2021a)
AFP1- I would feel proud
AFP2- I would feel accomplished
AFP3- I would feel confident
***Anticipated feeling of guilt*** (Han, 2014; Chi et al., 2021a)
AFG1- I would feel guilty
AFG2- I would feel remorseful
AFG3- I would feel sorry
***Attitude toward the pro-social behavior*** (Ajzen, 1991; Han and Hyun, 2017; Kim and Hwang, 2020)
APB1- For me, practicing pro-social behaviors at a heritage site by closely following the COVID-19 safe and healthy travel guidelines for my next vacation trip is – Foolish (1), Wise (7)
APB2- For me, practicing pro-social behaviors at a heritage site by closely following the COVID-19 safe and healthy travel guidelines for my next vacation trip is – Bad (1), Good (7)
APB3- For me, practicing pro-social behaviors at a heritage site by closely following the COVID-19 safe and healthy travel guidelines for my next vacation trip is – Unpleasant (1), Pleasant (7)
APB4- For me, practicing pro-social behaviors at a heritage site by closely following the COVID-19 safe and healthy travel guidelines for my next vacation trip is – Unfavorable (1), Favorable (7)
***Social norm*** (Han and Hyun, 2017; Kim and Hwang, 2020; Chi et al., 2021b)
SN1- Most people who are important to me think that for my next vacation trip I should practice pro-social behaviors by closely following the COVID-19 safe and healthy travel guidelines at a heritage site
SN2- For my next vacation trip, most people who are important to me would like me to practice pro-social behaviors by closely following the COVID-19 safe and healthy travel guidelines at a heritage site
SN3- People whose opinions I value would prefer that I practice pro-social behaviors for my next vacation trip by closely following the COVID-19 safe and healthy travel guidelines at a heritage site
***Pro-social behavioral intentions*** (Han and Hyun, 2017; Chi et al., 2021a)
PBI1- I plan to wear a mask at a heritage site for my next vacation trip
PBI2- I will exert effort in order to wear a mask at a heritage site for my next vacation trip
PBI3- I plan to keep my social distance from others at a heritage site for my next vacation trip
PBI4- I will exert effort in order to keep my social distance from others at a heritage site for my next vacation trip
PBI5- I plan to wash my hands frequently at a heritage site for my next vacation trip
PBI6- I will exert effort in order to practice handwashing properly at a heritage site for my next vacation trip
